# Transesophageal echocardiography in swine: evaluation of left and right ventricular structure, function and myocardial work

**DOI:** 10.1007/s10554-020-02053-7

**Published:** 2020-10-13

**Authors:** Sebastian Billig, Rashad Zayat, Andreas Ebeling, Henning Steffen, Christoph Nix, Nima Hatam, Heike Schnöring, Matthias Derwall

**Affiliations:** 1 University Hospital RWTH Aachen, Department of Anesthesiology, Medical Faculty, RWTH Aachen University, Pauwelsstr. 30, Aachen, 52074 Germany; 2 University Hospital RWTH Aachen, Department of Thoracic and Cardiovascular Surgery, Medical Faculty, RWTH Aachen University, Pauwelsstr. 30, Aachen, 52074 Germany; 3 Abiomed Europe GmbH, Neuenhofer Weg 3, Aachen, 52074 Germany

**Keywords:** Transesophageal echocardiography, Non-invasive hemodynamic monitoring, Speckle tracking, Myocardial work, Myocardial contractility, Swine model

## Abstract

This study aimed to determine standard left (LV) and right ventricular (RV) transesophageal echocardiographic (TEE) measurements in swine. Additionally, global myocardial work index (GWI) was estimated using pressure-strain loops (PSL). A comprehensive TEE examination was conducted in ten anesthetized, intubated and mechanically ventilated healthy female German landrace swine, weighing 44 to 57 kg. For GWI calculation, we performed LV and RV segmental strain analysis and used invasively measured LV and RV pressure to obtain PSL. The GWI and further myocardial work indices were calculated from the area of the PSL using commercially available software. Furthermore, hemodynamic measurements were obtained using indwelling catheters. We obtained complete standardized baseline values for left and right ventricular dimensions and function. Biplane LV ejection fraction was 63 ± 7 % and the LV end-diastolic volume was 70.5 ± 5.9 ml. Tissue Doppler estimated peak tricuspid annular systolic velocity was 13.1 ± 1.8 cm/s. The Doppler estimated LV and RV stroke volume index were 75.6 ± 7.2 ml/m^2^ and 76.7 ± 7.8 ml/m^2^ respectively. Pulsed wave Doppler derived cardiac output correlated well with cardiac output estimated using the thermodilution method (7.0 ± 1.2 l/min vs. 7.0 ± 1.1 l/min, r = 0.812, p = 0.004). The LV global longitudinal strain was -21.3 ± 3.9 % and the RV global longitudinal strain was -15.4 ± 2.5 %. LV GWI was 1885(1281–2121) mmHg*% and 297 ± 62 mmHg*% for the RV. LV global myocardial work efficiency was 82.6 ± 4 % and 83(72–88) % for the RV. TEE offers sufficient morphological, functional and hemodynamic assessment of the heart in swine. Myocardial contractility and mechanics can be reliably evaluated with the non-invasive GWI derived from echocardiography without additional invasive measures.

## Introduction

Echocardiography is an invaluable diagnostic tool for analysis of cardiac anatomy and function [1, 2]. Therefore, it is widely used in daily clinical routine as well as in research settings. Since the porcine cardiovascular system closely resembles human anatomy and physiology, swine are frequently used as animal models [3–5]. Echocardiography has been widely used in different porcine models such as myocardial infarction [6–10]. However, transthoracic echocardiography (TTE) in swine is associated with restrictions due to the keel-shaped thorax and narrow spaced ribs [11]. For instance, TTE can be limited in yielding reliable measurements to assess systolic and diastolic function, as apical views can frequently not be obtained in closed-chest swine models [7, 12, 13]. In contrast, transesophageal echocardiography (TEE) is free of these limitations [14, 15]. Here we report our findings regarding the use of TEE in swine. Only few publications have yet systematically described acquisition and results from porcine TEE-studies [14–16]. Moreover, data on right ventricular (RV) function and myocardial mechanics are lacking. Specifically, we aimed to determine the normal baseline values of standard two- dimensional (2D) and speckle-tracking echocardiography (2DSTE) measurements of the left ventricle (LV) and right ventricle (RV) in German landrace swine under general anesthesia. Baseline values of LV and RV dimensions, function and mechanics were determined using 2D imaging, Doppler modality, tissue Doppler and 2DSTE. Additionally, we applied a novel noninvasive method to calculate global myocardial work indices (GWI) using pressure-strain loops as described by Russel and colleagues [17–20].

## Methods

The experimental protocol was approved by the appropriate governmental institution (Landesamt für Natur, Umwelt und Verbraucherschutz Nordrhein-Westfalen, LANUV NRW). All animals received adequate care according to the precepts of the Helsinki Declaration of 1964 and its later amendments. Ten healthy female swine (Deutsche Landrasse, Sus scrofa Domestica) weighing 44 to 57 kg were used in this prospective study. The pigs were approximately four months of age. Veterinary inspection on arrival helped to ensure a consistently thorough health status. Animals were housed in pens with a 12 h-day-night cycle and access to drinking water ad libitum. Twelve hours prior to the experiment, animals were set on nil per os, except for drinking water access.

### Animal instrumentation

Weighing of the animals was performed before experiments were started. General anesthesia was induced by intramuscular injection of 4 mg/kg azaperone (Stresnil, Janssen-Cilag GmbH, Neuss, Germany), followed by intravenous injection of 15 mg/kg sodium pentobarbital (Narcoren, Boehringer Ingelheim Vetmedica GmbH, Ingelheim am Rhein, Germany). Anesthesia was preserved by continuous intravenous sodium pentobarbital infusion at a rate of 4 mg/kg/h. Placed in supine position, animals were orotracheally intubated and legs were carefully fixed in an extended position. Animals were ventilated with an inspiratory oxygen fraction of 0.3 (Servo Ventilator 300A; Siemens AG, Munich, Germany). Tidal volume was set to 10 ml/kg and respiratory rate was adjusted to keep the end-tidal carbon dioxide tension within a physiologic range (35 ± 4 mmHg). Continuous five lead electrocardiogram (ECG) and pulse oximetry were performed. Convective heating was used to maintain swine’s body temperature at 38.0 ± 0.5°C during preparation (Warm Touch 5200; Tyco Healthcare, Pleasanton, CA, USA). At completion of the protocol, anesthesia was stopped and animals were weaned from the ventilator.

### Hemodynamic monitoring

Arterial blood pressure was obtained using a fluid-filled catheter (Vygon, Ecquen, France), that was placed into the left femoral artery. For pulmonary artery pressure and cardiac output (CO_TD_) measurements by thermodilution, a Swan-Ganz catheter (744HF75; Edwards Lifesciences, Irvine, CA, USA) was flow-directed into the pulmonary artery and connected to a cardiac output monitor (Vigilance; Edward Lifesciences). When hemodynamic monitoring had been fully established, animals were left untreated for approximately thirty minutes. After this stabilization period, comprehensive baseline measurements were obtained.

### Echocardiography

Echocardiography was performed using a GE Vivid E9 system equipped with a 6VT-D (3.0–8.0MHz) TEE probe (GE Vingmed Ultrasound AS, Horten, Norway). Frames were recorded ECG-synchronized from at least three consecutive cardiac cycles and stored on a hard disk. Two experienced and certificated sonographers performed the TEEs and the offline data analysis. TEE studies and chamber quantification were performed according to the guidelines of the American Society of echocardiography and the European association of cardiovascular imaging [2, 21]. The data analysis including myocardial work estimation was carried out using the commercially available software EchoPAC (Version 202; GE Vingmed Ultrasound AS, Horten, Norway). EchoPAC provides validated assessment of LV myocardial work indices. To our knowledge, measurements for RV myocardial work indices with EchoPAC are not validated by GE healthcare or by any other research group yet.

For placement of the TEE-probe, a custom-made bite guard was inserted into the pig’s snout. The probe was protected by an ultrasound gel filled probe cover. Before probe introduction, we lubricated the coat’s outer surface with ultrasound gel. To avoid advancement into the porcine pharyngeal pouch, the tongue was lifted with a spatula, and the TEE probe was carefully inserted from the right side of the pig’s pharynx, applying minimum force [6, 22].

#### TEE views

**Fig. 1:**
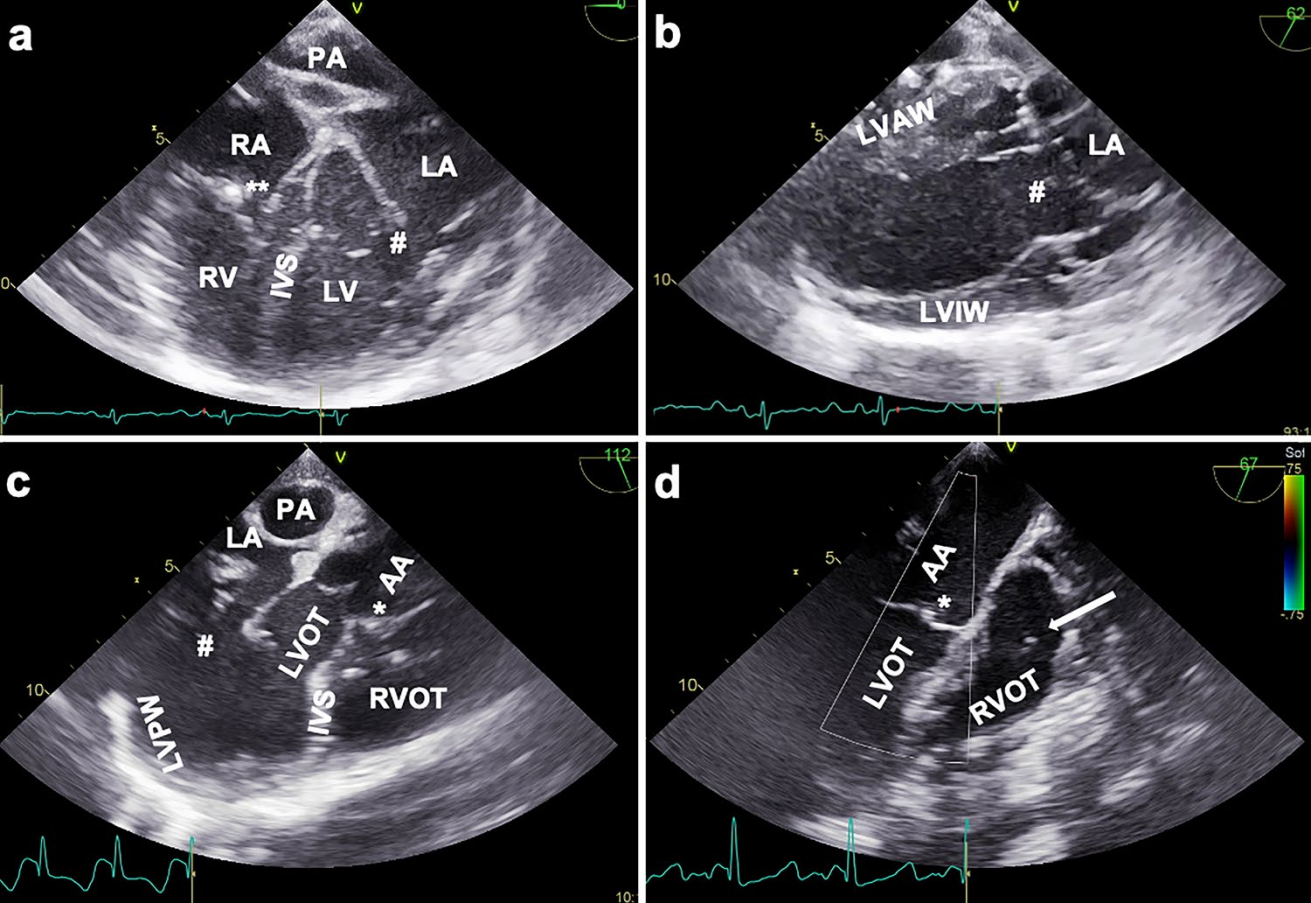
Representative TEE views in swine. **a** TEE mid-esophageal modified four-chamber view, array at 0°. *RA* right atrium, ** tricuspid valve, *RV* right ventricle, *IVS* interventricular septum, *PA* pulmonary artery, *LA* left atrium, # mitral valve, *LV* left ventricle. **b** TEE transgastric two-chamber view, array at 62°. *LVAW* left ventricular anterior wall, *LVIW* left ventricular inferior wall, *LA* left atrium, # mitral valve. **c** TEE mid-esophageal three-chamber view, showing the left ventricle in a long axis, array at 112°. *PA* pulmonary artery, *LA* left atrium, # mitral valve, *LVPW* left ventricular posterior wall, *LVOT* left ventricular outflow tract, * aortic valve, *AA* ascending aorta, *RVOT* right ventricular outflow tract, *IVS* interventricular septum. **d** TEE upper-oesophageal view of ascending aorta and aortic valve, array at 67°. *LVOT* left ventricular outflow tract, * aortic valve, *AA* ascending aorta, *RVOT* right ventricular outflow tract, *arrow* pulmonary valve

In general, anteflexion and lateroflexion of the probe was needed for unaffected imaging. Small adjustments in rotation, depth and flection of the probe were made in every animal to adapt to the individual anatomy. At 0–20° array position with slight retroflexion, the echocardiographic evaluation was started with a mid-esophageal modified four-chamber (4CH) view (Fig. 1a). In addition to the standard human four-chamber view, this plane allows for imaging of the pulmonary artery as well. From the mid-esophageal position, we assessed the ventricles in further views by modifying the transducer rotation: A two-chamber (2CH) view was generated at a beam rotation of 60–90° (Fig. 1b). To obtain the 2CH view a deep esophageal probe placement and even sometimes a transgastric position was needed. Further beam rotation to 100–130° resulted in a mid-esophageal three-chamber-view, showing a long axis (LAX) of the left ventricle and right ventricular outflow tract (RVOT) (Fig. 1c). An upper-esophageal view with 4–6 cm withdrawal of the probe from the mid-esophageal position and plane rotation of the transducer between 40° and 70° visualized the pulmonary and the aortic valve in a LAX (Fig. 1d). Further withdrawal of the probe resulted in a LAX in- and outflow view of the RV including the RV free wall.

#### Chamber quantification

LV dimensions were measured in the mid-esophageal 4CH view and deep esophageal 2CH view in systole and diastole. Left ventricular end-diastolic and end-systolic volumes (LVEDV, LVESV) were determined using biplane method of disk summation, also known as Simpson’s biplane method as recommended by ASE and EACVI [1].

RV basal diameter D_1_, mid diameter D_2_ and longitudinal diameter D_3_ were measured in the mid-esophageal modified 4CH view.

#### Systolic ventricular function

Ejection fraction (EF) was calculated using the modified biplane Simpson’s method.

$$\rm {EF}=\frac{\rm { left ventricular end diastolic volume }-\rm { left ventricular end systolic volume}}{\rm {left ventricular end diastolic volume}}\times 100$$

Cardiac output (CO_vol_) was calculated using the volumetric Simpson’s method. For calculation of the stroke volume index, body surface area (BSA) was calculated from the body weight using the following equation [3, 23]:

$$\rm {BSA}=0.0734\times {\rm { body weight}}^{0.656} $$

Color Doppler was applied in 4CH view to exclude any valvular pathologies.

Right ventricular fractional area change (RVFAC) and the tricuspid annular systolic velocity (TASV) were determined as indices of right ventricular function in the 4CH view. Endocardial right ventricular borders were traced in end-systole and end-diastole to obtain the corresponding RV areas. Subsequently, RVFAC was calculated [24]:

$$\text{RVFAC} = \frac{\rm { RV end diastolic area}-\rm {RV end systolic area}}{\rm {RV end diastolic area}}\times 100 $$

**Fig. 2:**
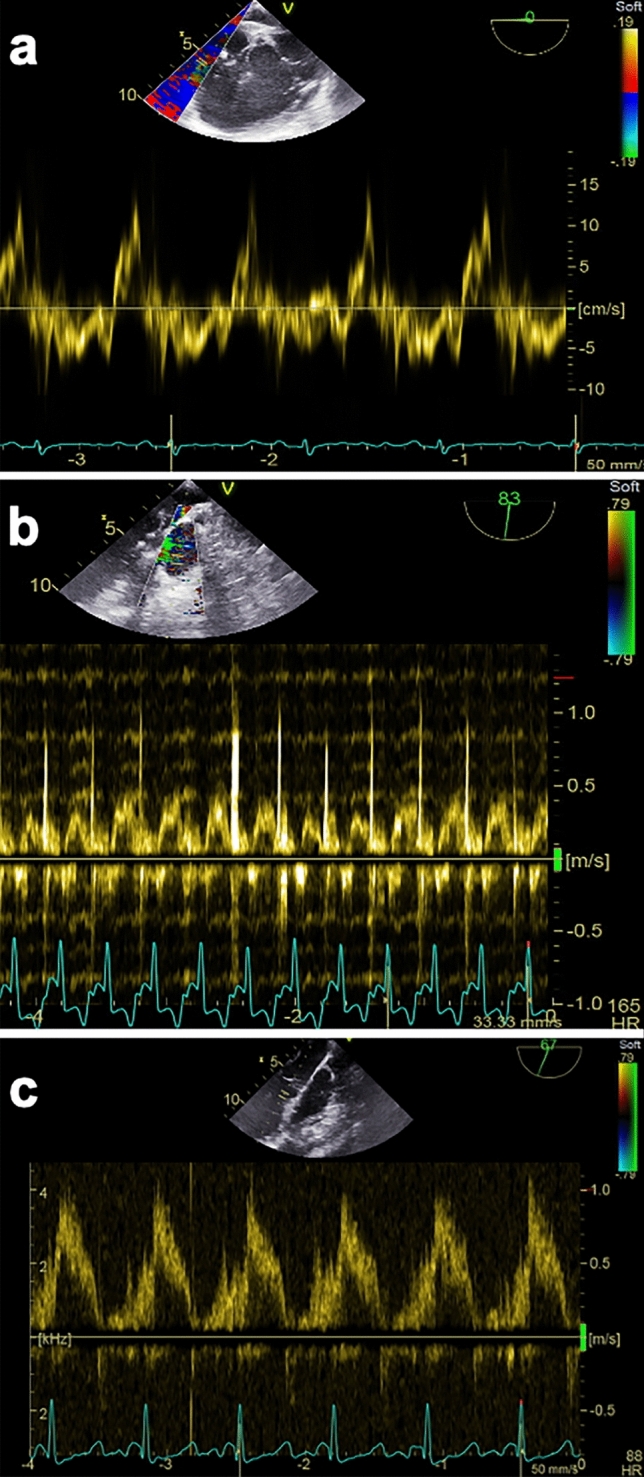
Representative TEE pulsed wave Doppler and tissue Doppler measurements. **a** Tissue Doppler beam aligned with the lateral tricuspidal annulus to measure tricuspidal annular plane systolic velocity (TASV). **b** TEE pulsed wave Doppler measurement in the right ventricular outflow tract. **c** TEE pulsed wave Doppler measurement in the left ventricular outflow tract

TASV was captured in 4CH views at the intersection of the RV free wall and the anterior leaflet of the tricuspid valve using tissue Doppler (Fig. 2a) [25].

#### Hemodynamics

Pulsed wave Doppler (PWD) was applied in the LAX-views of the pulmonary and the aortic valve to obtain the corresponding velocity time integrals (VTI) from the left (LVOT) and right (RVOT) ventricular outflow tract (Fig. 2b and c). Upper esophageal LAX-views provided sufficient angle alignment between blood flow and Doppler ultrasound transducer. Ultrasound derived Cardiac output (CO_PWD_) was computed using the following equations [26].

$${\rm {CO}}_{\rm {PWD pulmonary}}=\rm {RVOT VTI}\times {\rm {cross}-\rm {sectional area}}_{\rm {RVOT}}\times \rm {heart rate}$$

$${\rm {CO}}_{\rm {PWD aortic}}=\rm {LVOT VTI}\times {\rm {cross}-\rm {sectional area}}_{\rm {LVOT}}\times \rm {heart rate}$$

CO was divided by the heart rate to obtain the pulmonary and aortic stroke volume (SV).

#### Deformation imaging and myocardial work analysis

Myocardial deformation imaging using 2DSTE offers an advanced LV quantification beyond ejection fraction (EF) [19]. However, 2DSTE is load-dependent and therefore changes in afterload can affect the diagnostic value of 2DSTE [19]. In 2012, Russell et al. [18] introduced a novel non-invasive method to calculate global myocardial work index (GWI) using pressure-strain loops. This novel non-invasive myocardial work parameter takes afterload as well as deformation into account and is therefore considered superior to 2DSTE as it offers additional insights into myocardial function [27].

**Fig. 3:**
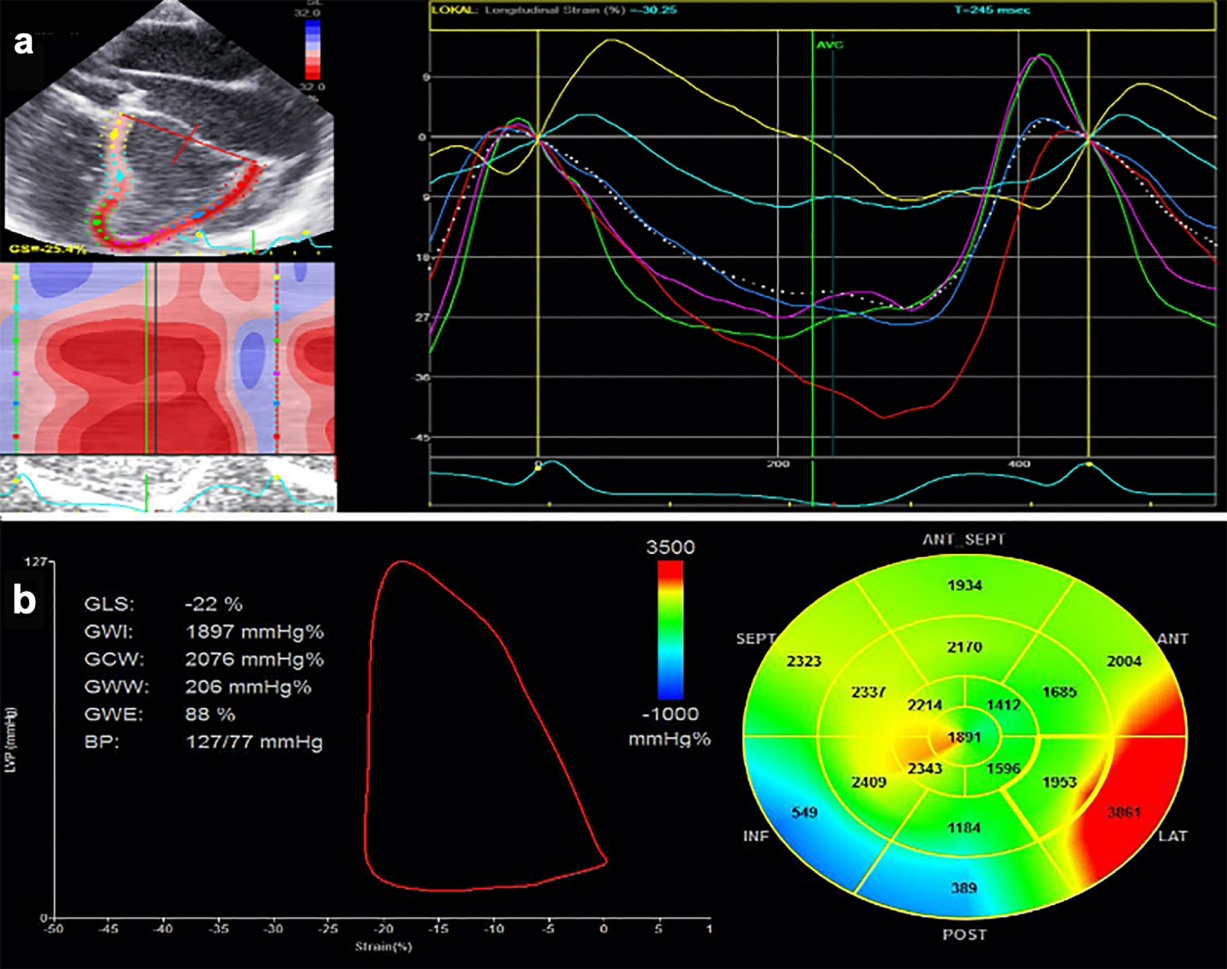
Exemplary demonstration of speckle trasssscking and myocardial work analysis. **a** Analysis of left ventricular peak systolic longitudinal strain obtained from a modified apical 4-chamber view with parametric (color-coded) display of end-systolic strain (left upper panel); M-mode representation of peak systolic strain (left lower panel); and strain-time curves: each colored curve displays a different segmental strain while the dotted white curve represents the average peak systolic longitudinal strain (right upper panel). **b** myocardial work indices obtained from pressure-strain loops with the myocardial work curve and the Bull’s eye representing segmental myocardial work indices. *GLS* global longitudinal strain, *GWI* global myocardial work index, *GCW* global constructive work, *GWW* global wasted work, *GWE* global effective work, *BP* blood pressure

**Fig. 4:**
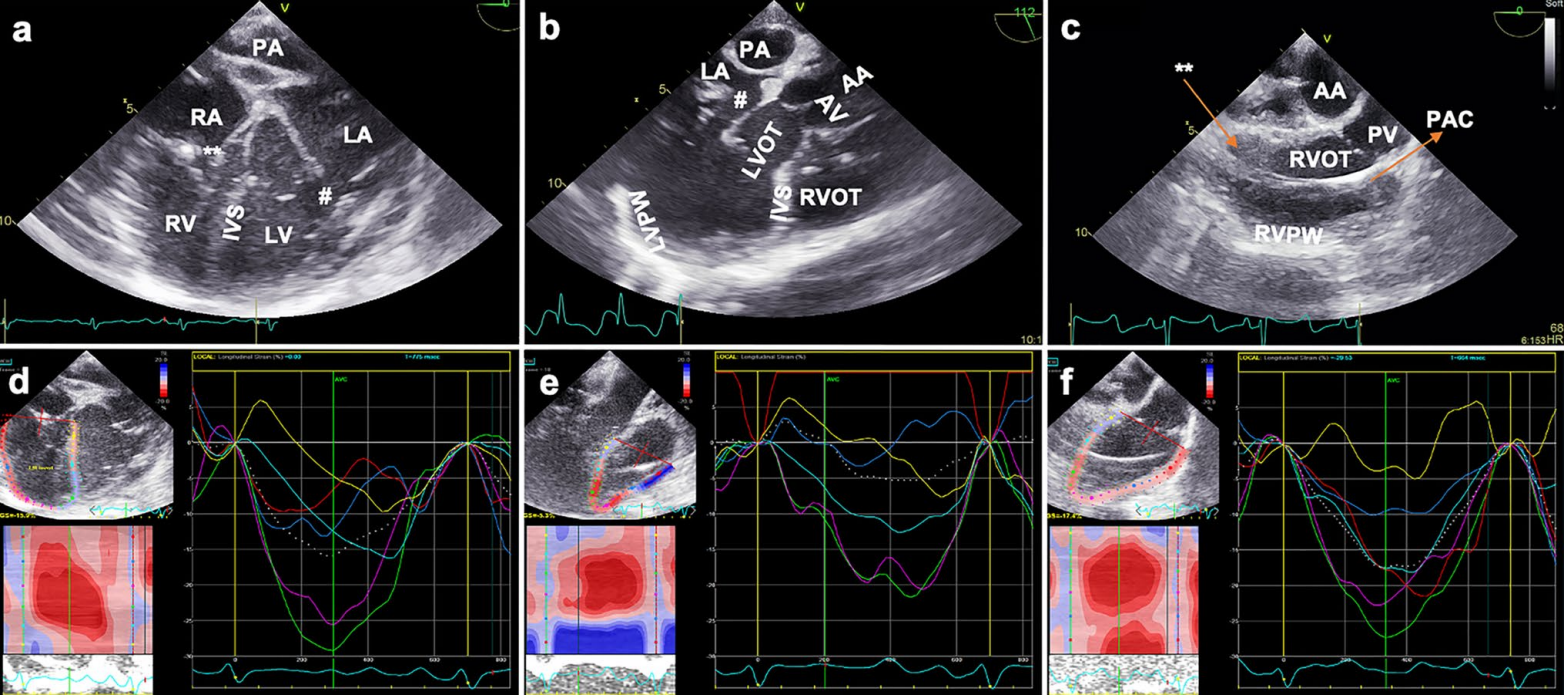
Right ventricular myocardial work assessment. **a** 4CH view for the RV anterior wall at 0°. *RA* right atrium, ** tricuspid valve, *RV* right ventricle, *IVS* interventricular septum, *PA* pulmonary artery, *LA* left atrium, # mitral valve, *LV* left ventricle. **b** modified upper LAX RV in- and outflow view showing the RV free wall at 112°. *PA* pulmonary artery, *LA* left atrium, # mitral valve, *AV* aortic valve, *AA* ascending aorta, *LVOT* left ventricular outflow tract, *IVS* interventricular septum, *RVOT* right ventricular outflow tract, *LVPW* left ventricular posterior wall. **c** mid-esophageal LAX RVOT view showing the posterior part of the RVOT at 0°. *AA* ascending aorta, *RVOT* right ventricular outflow tract, *RVPW* right ventricular posterior wall, ** tricuspid valve, *PAC* pulmonary artery catheter, *PV* pulmonary valve. **d** peak systolic longitudinal strain analysis of the anterior right ventricular obtained from a 4CH view (corresponding to panel a). **e** peak systolic longitudinal strain analysis of the right ventricular free wall obtained from a modified upper LAX RV in- and outflow (corresponding to panel b). **f** peak systolic longitudinal strain analysis of the right ventricular posterior part of the RVOT (corresponding to panel c). Panels (d–f): parametric (color-coded) display of end-systolic strain (left upper panel); M-mode representation of peak systolic strain (left lower panel); and strain-time curves: each colored curve displays a different segmental strain while the dotted white curve represents the average peak systolic longitudinal strain (right panel).

Before performing 2DSTE, aortic and mitral valve opening and closure time were determined by pulsed-Doppler from the mid-esophageal LAX view. Deformation imaging of the LV was applied in the mid-esophageal modified 4CH-, LAX- and deep-esophageal 2CH views (Fig. 3a). To perform 2DSTE analysis of the RV we used the 4CH view for the RV anterior wall (Fig. 4a), the modified upper LAX RV in- and outflow view showing the RV free wall (Fig. 4b) and the mid-esophageal LAX RV view showing the posterior part of the RVOT (Fig. 4c). 2DSTE was used to define LV and RV global longitudinal strain (GLS) [28]. The systolic arterial and pulmonary blood pressure was used to produce pressure-strain loops in EchoPAC (Fig. 3b). Left ventricular pressure is estimated by adjusting a reference pressure curve with a measured blood pressure and with echocardiography derived valvular event timing [17, 18]. We used the blood pressure derived from a femoral arterial line for the LV, and the pulmonary artery pressure derived from a Swan-Ganz catheter for the RV.

EchoPAC software creates an adjusted LV pressure curve corresponding to the duration of isovolumic and ejection phases determined by valvular timing events. As described by Russel et al. [17, 18], the area within the PSL delivers the index of the GW. The following parameters can then be calculated:

1. GWI: the total area of the PSL represents the total work from mitral valve closure to mitral valve opening.
2. Constructive MW (GCW): work of the LV, which contributes to the LV ejection during systole. Constructive MW is defined as shortening of the myocytes during systole. GCW = positive Work during systole + negative work during isovolumic relaxation.
3. Wasted MW (GWW): Work of the LV that does not contribute to the LV-ejection. Wasted MW is defined as lengthening of the myocytes (instead of shortening) during systole, which further intensifies the shortening during the isovolumic relaxation phase. GWW = negative work during systole + positive work during isovolumic relaxation.
4. MW Efficiency (GWE): is the fraction of constructive MW to total work and calculated by GCW/(GCW + GWW) [17, 18].

### Analysis

We used SPSS statistics Version 26 (IBM, Armonk, NY, USA) for data analysis. Normal distribution was confirmed using Kolmogorov–Smirnov test. Normal distributed measurements are expressed as mean ± standard deviation. If normal distribution was not present, data are expressed as median followed by range in brackets. To test for correlation between CO derived from indwelling catheters and echocardiographic measurements, we ran a Pearson correlation test. A *p* value smaller than 0.05 was considered significant.

## Results

We carried out a comprehensive TEE examination in 10 healthy female swine with a median body weight of 46.1 (43.6–56.7) kg. Study acquisition took on average between 20 and 30 min per animal. No adverse events concerning TEE probe insertion or TEE-study acquisition in general were observed. All the above-described views and structures could be visualized in each animal. No preexisting cardiac conditions were found in any of the examined animals.

### Chamber quantification

LVEDV was 70.7 ± 4.4 ml and LVESV was 32.6 ± 3.8 ml. RV basal diameter D_1_ was 3.3 ± 0.4 cm, mid diameter D_2_ was 2.5 (2.4–3.1) cm and longitudinal diameter D_3_ was 4.4 ± 0.2 cm.

### Systolic ventricular function

RVFAC was 44 ± 6 % and TASV was 13.1 ± 1.8 cm/s. General physiological parameters as well as TEE data about ventricular size and function are presented in Table 1. LV SV was 70.5 ± 5.9 ml and the biplane EF was 63.1 ± 7.1 %. BSA adjusted SV index was 75.6 ± 7.2 ml/m^2^ for LV and 76.7 ± 7.8 ml/m^2^ for RV.

**Table 1:** General data and TEE findings in swine and humans.

Parameter	Swine mean	SD/(range)	Mean human	Human SD
Weight (kg)	46.1	(43.6–56.7)		
Body surface area (m^2^)	0.91	(0.88–1.04)		
Heart rate (min^-1^)	92	± 13		
Swan-Ganz catheter derived cardiac output (l/min^-1^)	7.03	± 1.1		
Right ventricular dimensions and function				
Right ventricle basal diameter D_1_ (cm)	3.3	± 0.4	3.3^a^	± 0.4^a^
Right ventricle mid diameter D_2_ (cm)	2.5	(2.4–3.1)	2.7^a^	± 0.4^a^
Right ventricle longitudinal diameter D_3_ (cm)	4.4	± 0.2	7.1^a^	± 0.6^a^
Right ventricular end diastolic area (cm^2^)	10.5	± 1.6		
Right ventricular end systolic area (cm^2^)	5.9	± 0.9		
Right ventricular fractional area change (%)	44	± 6	49^a^	± 7^a^
Tricuspid annular systolic velocity (cm/s)	13.1	± 1.8	14.1^a^	± 2.3^a^
Pulmonal cardiac output (l/min)	7.0	± 1.2		
Pulmonal stroke volume (ml)	71.6	± 7.3		
Pulmonal stroke volume index (ml/m^2^)	76.7	± 7.8		
Left ventricular dimensions and function				
Left ventricular end diastolic volume (ml)	70.7	± 4.4	76^b^	± 15^b^
Left ventricular end systolic volume (ml)	32.6	± 3.8	28^b^	± 7^b^
Left ventricular ejection fraction (%)	63.1	± 7.1	64^b^	± 5^b^
Aortic cardiac output (l/min)	6.8	± 1.1		
Aortic cardiac index (l/m^2^)	7.35	± 1.2	4.41^c^	
Aortic stroke volume (ml)	70.5	± 5.9		
Aortic stroke volume index (ml/m^2^)	75.6	± 7.2		

Values are expressed as mean ± standard deviation. Data in brackets represent median and range of not normal distributed data. Selected human values are presented for reference

^a^Data reported in [31]

^b^Data reported in [1], values are presented for a female group

^c^Data reported in [48]

### Deformation imaging and myocardial work analysis

GLS was -21.3 ± 3.9% for LV and − 15.4 ± 2.5% for RV. GWI was 1885 (1281-2121) mmHg*% for LV and 297 ± 62 mmHg*% for RV. GCW was 2100 ± 421 mmHg*% for LV and 322 ± 84 mmHg*% for RV. Those values are comparable to values found in humans (Table 2). LV GWW was 360 ± 151 mmHg*% and 59 ± 11 mmHg*% for RV. GWE was 82.6 ± 4% for the LV and 83(72–88) % for the RV. LV GWW was about four-fold higher in swine then in men translating into a lower LV GWE in swine than in men (82.6 ± 4%) in swine vs. 96 (94–97)% in men.

**Table 2:** TEE derived myocardial work values in pigs and humans

parameter	Swine mean	SD/(range)	Human mean	Human SD/(range)
Left ventricular mechanics				
Systolic blood pressure (mmHg)	128	± 17	116^a^	± 12^a^
Diastolic blood pressure (mmHg)	68	± 6	73^a^	± 8^a^
Global longitudinal strain (%)	− 21.3	± 3.9	− 21.2^b^	± 2.4^b^
Global work index (mmHg*%)	1885	(1281–2121)	1896^a^	± 308^a^
Global constructive work (mmHg*%)	2100	± 421	2232^a^	± 331^a^
Global wasted work (mmHg*%)	360	± 151	78.5^a^	(53–122.2)^a^
Global myocardial work efficiency (%)	82.6	± 4	96^a^	(94–97)^a^
Right ventricular mechanics				
Systolic pulmonal arterial pressure (mmHg)	26	± 3		
Diastolic pulmonal arterial pressure (mmHg)	13	± 3		
Global longitudinal strain (%)	− 15.4	± 2.5		
Global work index (mmHg*%)	297	± 62		
Global constructive work (mmHg*%)	322	± 84		
Global wasted work (mmHg*%)	59	± 11		
Global myocardial work efficiency (%)	83	(62–88)		

Data are expressed as mean ± standard deviation. Data in brackets represent median and range of not normal distributed data

^a^Data reported in [44]

^b^Data reported in [1]

### Hemodynamics

Mean heart rate during the examinations was 92 ± 13 beats per minute. Mean arterial blood pressure was 128 ± 17 mmHg systolic and 68 ± 6 mmHg diastolic. Mean pulmonary artery pressure was 26 ± 3 mmHg systolic and 13 ± 3 mmHg diastolic. PW-Doppler estimated LV SV was 70.5 ± 5.9 ml and SV indexed to BSA was 75.6 ± 7.2 ml/m^2^ for LV and 76.7 ± 7.8 ml/m^2^ for RV.

CO was evaluated by three different methods. Thermodilution-method derived CO_TD_ was 7.0 ± 1.1 l/min. PWD derived pulmonary CO_PWD_ was 7.0 ± 1.2 l/min and PWD derived aortic output was 6.8 ± 1.1 l/min. CO_vol_ calculated by the volumetric Simpson’s method was 5.1 ± 1.1 l/min.

**Fig. 5:**
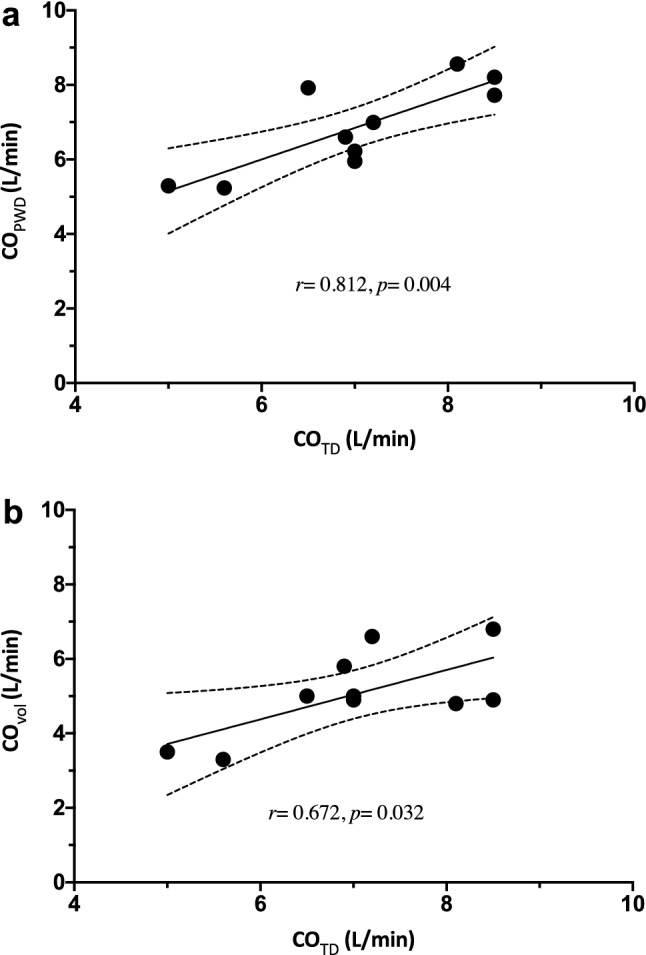
Correlation plot of thermodilution, pulsed wave Doppler and volumetric derived cardiac output. Invasively obtained cardiac output measured by pulmonary artery catheter correlated well with TEE measurements. **a** Comparison of CO_TD_ and CO_PWD_ **b** Comparison of CO_TD_ and CO_vol_. CO_TD_ = cardiac output determined using thermodilution via a Swan-Ganz catheter, CO_PWD_ = Ultrasound derived Cardiac output using pulsed wave Doppler, CO_vol_ = Ultrasound derived Cardiac output using volumetric calculations. Dotted lines represent 95%-confidence interval

A linear regression analysis and pearson’s correlation were performed to compare the estimated CO between the three methods (Fig. 5). Invasively measured CO_TD_ correlated well with both the PWD derived CO_PWD_ (*r* = 0.812, *p* = 0.004) and the volumetric estimated CO_vol_ (*r* = 0.672, *p* = 0.032).

## Discussion

The study at hand is unique in its capability to demonstrate both feasibility and reliability of sophisticated TEE based measurements in anesthetized swine. To the best of our knowledge, this is the first trial to evaluate LV and RV myocardial function and mechanics using the novel none-invasive myocardial work estimation method by Russel and colleagues in this setting. Moreover, our data not only complement porcine standard values that have been determined by others [29, 30], but furthermore adds novel insight into myocardial work of both ventricles in swine.

These observations are significant due to the importance of porcine animal models of cardiovascular disease. Swine are frequently chosen as model-organism for cardiovascular disease due to their close resemblance of human anatomy and physiology [3–5]. More specifically, our findings show that LV GLS in swine is comparable to LV GLS in man. LV GWI and GCW were also comparable to standard values derived from human data. Conversely, global wasted work was found to be four times higher in swine with 360 mmHg*% vs. 78.5 mmHg*% in men. One possible explanation is that our measurements were carried out in anesthetized swine, while human reference values were derived from TTE in awake healthy subjects. Hence, anesthesia drugs and positive pressure ventilation appear to have shifted the myocardial wasted work ratio towards negative work in our setting.

Our RV data about TASV and the RVFAC appear to resemble findings in humans [1, 31]. In terms of dimensions, it is noticeable that RV basal and mid diameters D_1_ and D_2_ were comparable in swine and humans, while the longitudinal diameter D_3_ was shorter in swine [31]. By and large, the measured LV parameters were comparable to that in humans. The EF was similar, while CO was found to be higher in swine than in humans. However, other studies in swine found similar values regarding CO and RV/LV volumes [15, 29–32]. As swine are known to compensate for a low blood hemoglobin level with a high CO in order to achieve a sufficient oxygen tissue supply [33], the higher CO may be a result of structural and physiological differences between the species.

Comparing our data to the porcine TEE study of Huenges et al. we noticed a similar EF in conjunction with half the CO found in our experiments [14], despite being estimated in equally sized animals. These differences in CO values may be based on differences in the anesthesia regimen. Furthermore, CO is calculated using an LVOT cross sectional area that is prone to misestimation.

Validation of our echo-obtained CO data with an invasively measured reference method [26] vastly improved the credibility of our results. We observed a strong positive correlation of cardiac output measured by thermodilution with PWD derived values. This correlation implies a good accuracy of our echocardiographic measurements. The correlation between volumetric estimated CO_vol_ and the thermodilution CO_TD_ was good as well, but lower than between CO_PWD_ and CO_vol_. One possible explanation for the discrepancy between CO_PWD_ and CO_vol_ is the difficulty to obtain the correct anatomical 2-CH view and the 4-CH leading to underestimated values due to measurements in foreshortened views.

Certain limitations apply to the results gained in our study. First, study results were obtained in a fairly small sample size of 10 animals. However, choosing a small sample size is particularly desirable from an ethical perspective wherever results are valid. Second, TEE measurements were performed in swine anaesthetized with pentobarbital. Previous research revealed conflicting data on pentobarbital effects on hemodynamics as compared to awake animals [34–37]. However, all known anesthetics have significant impact on hemodynamic properties [38], and acquisition of TEE-studies in swine is next to impossible without sedation. Third, imaging the apex is tremendously difficult in swine. Attention was paid to avoid foreshortening of the ventricles in echocardiography and ante- and retroflexion of the tip was used to find the appropriate plane of the heart. However, foreshortening of the ventricle might have caused an underestimation of the concerning ventricular volumes. 3D-TEE is believed to overcome these limitations and would probably have led to a more precise calculation of derived parameters [26]. Other important limitations are imposed by the RV myocardial work indices used here. To the best of our knowledge, no specific software for non-invasive RV myocardial work assessment is commercially available at this time. Furthermore, peer-reviewed publications on comparisons of TEE-derived pressure-strain measurements versus invasive pressure-volume loops are lacking. In our analysis, we used commercially available LV software provided by GE. Therefore, results from our RV myocardial work studies must be interpreted with caution. Furthermore, image quality was limited in a few cases. These limitations are due to specific anatomical features of swine in which the main bronchus may interleave between heart and esophagus [15, 16]. Another limitation concerning the MW measurements is a possible difference between central arterial systolic pressure and femoral arterial systolic pressure. Central pressure might have been lower than peripheral pressure due to pressure augmentation. Heart rate variability and arrhythmia, with significant beat-to-beat variability affect the accuracy and reliability of 2DSTE assessments with the viability of MW estimation. However, significant arrhythmia was not observed in any of the examined animals. Animals were scheduled to undergo a separate experimental protocol following the echocardiographic assessments described in this manuscript. As this protocol included weaning of the pigs from the ventilator, invasive measures were kept to an absolute minimum in order to reduce the risk for procedure related adverse events. Hence, we failed to determine left (LVP) and right ventricular pressure (RVP) that would have allowed for plotting of true pressure-volume loops. However, doing so will be an additional asset that will be incorporated into future experimental protocols.

Compared to TTE, TEE offers significant advantages in swine: The close anatomic relation of the esophagus to the heart enables high resolution images of the heart. TEE can be applied in the closed chest without significant trauma. This can be paramount in chronic animal experiments. In addition, TEE may be favorable in open-chest models, as it allows for echocardiographic assessment without interfering with surgical interventions. If longitudinal strain values are to be measured, TEE might also be the superior choice over TTE, as apical views may not be achievable using transthoracic echocardiography. However, for the acquisition of longitudinal strain calculation, apical planes are critical [12, 39].

In general, an important constraint in interpretation of conventional strain data is the load dependency [19, 28]. Afterload-dependence of the strain must be considered especially in settings in which large fluctuations of the afterload are likely to occur. We used a method described by Russel et al. that is more robust to afterload changes than strain with regards to described systolic function. This method computes work with strain imaging and an estimated left ventricular pressure [19, 20]. The calculated myocardial work reflects myocardial oxygen-consumption and metabolic rate [18, 40, 41]. This methodology has been employed in human clinical research before [19, 20, 42–44]. Transferring this method to animal experiments opens up new options for translational research: For example, the impact of new drugs on myocardial work can be studied semi-invasive by TEE in chronic experiments. This method might also be used in the context of cardiac stimulation research. Cardiac resynchronization therapy has been evaluated in humans using Russel’s method by analyzing the wasted myocardial work before and after implantation of a resynchronization device [45, 46]. The efficacy of novel cardiac stimulation systems might therefore be studied with TEE in a porcine model of heart failure in the future. Furthermore, consideration of the performed myocardial work is crucial in the evaluation of ventricular assist devices, as they are supposed to reduce heart work. Research on left ventricular assist devices with the help of echocardiographic assessed myocardial work is likely to bring new insights into this technology, especially as the strategies presented here may be employed to monitor RV function that is known to be critical to assist device function [47].

## Conclusions

TEE offers sufficient morphological, functional and hemodynamic assessment of the heart in swine. The non-invasive GWI may offer an improved assessment of myocardial contractility and mechanics. The established baseline values will inform design and validation of future studies in translational cardiovascular research.

## Data Availability

The authors declare that they have full control of all primary data and that they agree to allow the journal to review their data if requested.
